# Advances in intrahepatic and extrahepatic vascular dysregulations in cirrhotic portal hypertension

**DOI:** 10.3389/fmed.2025.1515400

**Published:** 2025-01-31

**Authors:** Yanqiu Li, Bingbing Zhu, Ke Shi, Yu Lu, Xuanwei Zeng, Yongqi Li, Qun Zhang, Ying Feng, Xianbo Wang

**Affiliations:** Center for Integrative Medicine, Beijing Ditan Hospital, Capital Medical University, Beijing, China

**Keywords:** cirrhotic portal hypertension, sinusoidal capillarization, endothelial dysfunction, splanchnic vasodilation, hyperdynamic circulation, nitric oxide

## Abstract

Cirrhotic portal hypertension, the most prevalent and clinically significant complication of liver cirrhosis, manifests as elevated portal venous pressure and is associated with severe complications. Although much research on the mechanisms of portal hypertension has focused on liver fibrosis, less attention has been given to the role of intrahepatic and extrahepatic vascular dysfunction, particularly with respect to extrahepatic vasculature. While the role of hepatic fibrosis in cirrhotic portal hypertension is undeniable, the underlying mechanisms involving intrahepatic and extrahepatic vasculature are highly complex. Sinusoidal capillarization and endothelial dysfunction contribute to increased intrahepatic vascular resistance. Hemodynamic changes in the extrahepatic circulation, including splanchnic vasodilation and hyperdynamic circulation, play a significant role in the development of portal hypertension. Additionally, therapeutic strategies targeting these vascular mechanisms are diverse, including improvement of sinusoidal microcirculation, therapies targeting hepatic stellate cells activation, and pharmacological modulation of systemic vascular tone. Therefore, in this review, we will discuss the vascular-related mechanisms and treatment progress of portal hypertension in cirrhosis to provide a new theoretical basis and practical guidance for clinical treatment.

## 1 Introduction

Liver cirrhosis is pathologically characterized by hepatocyte necrosis, fibrous tissue proliferation, and intrahepatic vascular remodeling (1). Portal hypertension is one of the most common and severe complications of cirrhosis, significantly impacting patients’ quality of life and prognosis (2). Except for fibrosis, the mechanisms underlying portal hypertension are complex, involving both intrahepatic and extrahepatic factors. The development of portal hypertension is primarily attributed to increased intrahepatic vascular resistance and increased portal venous inflow. Increased intrahepatic vascular resistance arises from structural changes induced by hepatic fibrosis and regenerative nodule formation. Additionally, the activation of hepatic stellate cells (HSCs) and the excessive proliferation of myofibroblasts play crucial roles (3). The activation of these cells not only elevates intrahepatic vascular resistance but also promotes further fibrosis through the release of various cytokines and growth factors (4). Moreover, endothelial dysfunction and microvascular structural alterations significantly impact hepatic hemodynamics (5). The anticoagulant properties of liver sinusoidal endothelial cells are diminished, promoting thrombosis, which further exacerbates portal hypertension (6). Extrahepatically, vascular remodeling of the portal venous system and the formation of portosystemic collaterals are important compensatory mechanisms (7). While these vascular changes can partially alleviate pressure within the portal system, they can also lead to serious complications such as esophageal and gastric varices and gastrointestinal bleeding.

However, the intrahepatic and extrahepatic vascular regulatory mechanisms governing portal hypertension are complex and a comprehensive understanding remains elusive. Current therapeutic options, while capable of reducing portal pressure and preventing bleeding to some extent, have limited efficacy and are associated with side effects. Therefore, this review aims to systematically summarize the intrahepatic and extrahepatic vascular mechanisms of portal hypertension in liver cirrhosis, analyze the progress of existing research and explore potential therapeutic strategies and future research directions. Through a comprehensive understanding of the pathophysiological mechanisms of portal hypertension, we expect to provide a theoretical basis and novel insights for clinical management, thereby improving the prognosis and quality of life for patients with cirrhosis.

## 2 Intrahepatic vascular changes in cirrhotic portal hypertension

**Figure 1:**
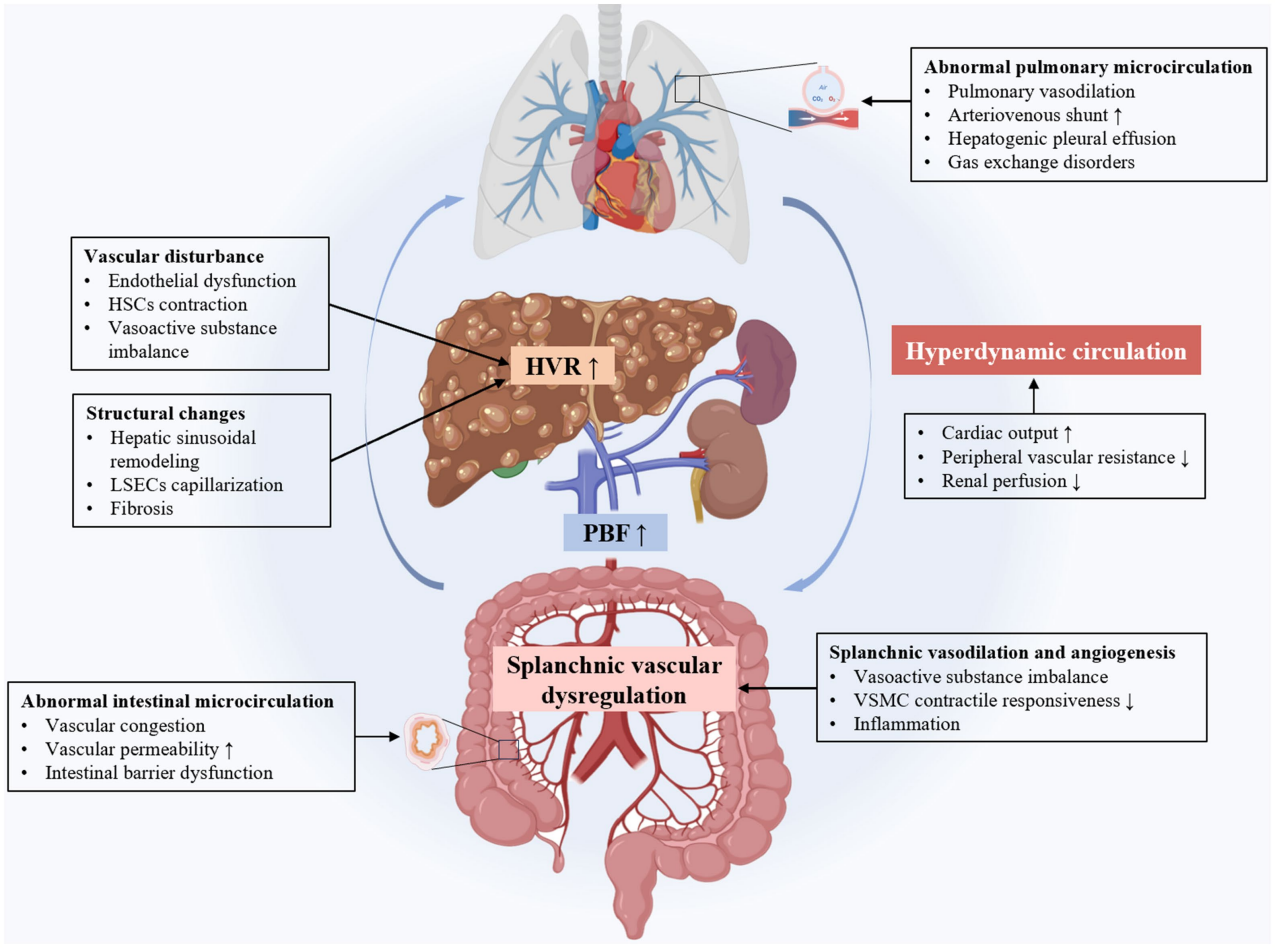
The intrahepatic and extrahepatic mechanisms of cirrhotic portal hypertension. LSECs, liver sinus endothelial cells; HSCs, hepatic stellate cells; HVR, hepatic vascular resistance; PBF, portal blood flow; VSMC, vascular smooth muscle cell.

During the process of cirrhosis, the liver’s internal vasculature undergoes significant structural changes, mainly manifested as sinusoidal remodeling and capillarization. In terms of functional changes, endothelial cell damage leads to reduced nitric oxide (NO) synthesis, imbalance of systolic and diastolic vascular factors, and increased blood flow resistance, ultimately leading to the occurrence and development of portal hypertension (Figure 1).

### 2.1 Intrahepatic structural changes

**Figure 2:**
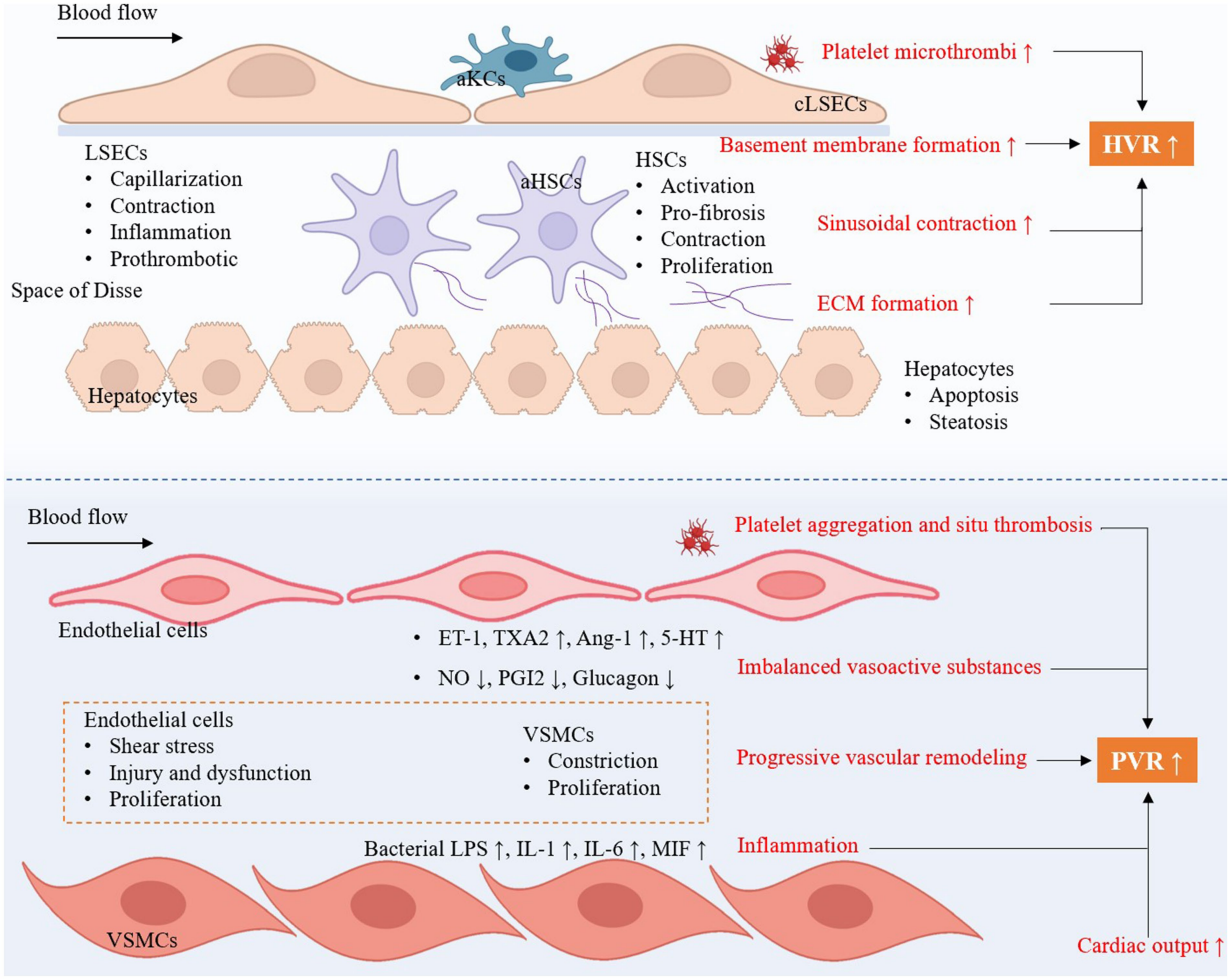
The key intrahepatic cellular changes occur in cirrhotic portal hypertension and the development of portopulmonary hypertension. HVR, hepatic vascular resistance; PVR, pulmonary vascular resistance; aKCs, activated Kupffer cells; LSECs, liver sinus endothelial cells; HSCs, hepatic stellate cells; aHSCs, activated hepatic stellate cells; cLSECs, capillarized liver sinus endothelial cells; ECM, extracellular matrix; ET-1, endothelin-1; TXA2, thromboxane A2; Ang-1, angiotensin-1; 5-HT, 5-Hydroxytryptamine; NO, nitric oxide; PGI2, prostaglandins I2; VSMCs, vascular smooth muscle cells; LPS, lipopolysaccharides; IL-1, interleukin-1; IL-6, interleukin-6; MIF, macrophage migration inhibitory factor.

During the development of liver cirrhosis, the intrahepatic vascular system undergoes significant structural changes. Major structural changes include hepatic sinus remodeling and capillarization. Hepatic sinuses are special capillary-like structures. Liver sinus endothelial cells (LSECs) in hepatic sinuses have fenestrae that allow direct contact between blood and hepatocyte, facilitating the exchange of substances (5). In liver cirrhosis, hepatic sinus remodeling and capillary vascularization are key pathophysiological changes and affect intrahepatic hemodynamics. Hepatic sinus remodeling involves LSECs dysfunction, capillarization, and activation of Kupffer cells and HSCs (Figure 2).

LSECs dysfunction and capillarization play a role in hepatic sinus remodeling. LSECs maintain important physiological functions in healthy liver, including regulation of liver blood flow, substance exchange, and immune surveillance (5, 8). In liver cirrhosis, LSECs lose their normal fenestrae structure and form a continuous basement membrane, a process known as hepatic sinusoidal capillarization (4, 5). Capillary vascularization leads to increased blood flow resistance and increased portal vein pressure. The formation of basement membrane is another key link in the hepatic sinusoidal capillarization. Dysfunctional LSECs secrete large amounts of basement membrane components, such as type IV collagen and laminin (9). These components are deposited beneath the LSECs, forming a continuous basement membrane (5). This basement membrane increases blood flow resistance and impedes the exchange of substances. Besides, dedifferentiation of LSECs means LSECs lost their characteristic fenestrated structure (10). This change causes the hepatic sinuses to lose their high permeability, obstructing material exchange and increasing blood flow resistance (11). The reasons for the loss of fenestration include endothelial cell damage, chronic inflammatory response and persistent cytokine stimulation.

Activation of HSCs play a central role in hepatic sinus remodeling and capillary vascularization. In liver cirrhosis, HSCs change from a resting state to an active state, similar to myofibroblasts, producing large amounts of extracellular matrix (ECM) (12, 13). These ECM components further promote the formation of basement membrane and hepatic sinusoidal capillarization. HSCs also release a variety of pro-fibrotic factors, such as transforming growth factor-*β* (TGF-β) and platelet-derived growth factor (PDGF), which further promotes the fibrotic process and hepatic sinus remodeling (14).

Kupffer cells are resident macrophages in liver that play important immune and clearance functions in the hepatic sinuses (15). In liver cirrhosis, Kupffer cells are activated and release multiple inflammatory mediators and chemokines, such as tumor necrosis factor (TNF-*α*), interleukin-6 (IL-6), and reactive oxygen species (ROS) (16). These inflammatory mediators not only cause local inflammatory response, but also promote the activation of HSCs and the dysfunction of LSECs (17). It also causes hepatocytes apoptosis and necrosis by releasing ROS and cytokines, and exacerbating hepatic sinus remodeling and fibrosis.

### 2.2 Intrahepatic functional changes

#### 2.2.1 Endothelial dysfunction

Endothelial cell damage occurs in the early stages of cirrhosis and portal hypertension. Chronic inflammation and oxidative stress are the main factors leading to endothelial cell damage in liver cirrhosis (18). Chronic hepatitis, alcoholic liver disease, non-alcoholic fatty liver disease (NAFLD) and other causes can cause persistent inflammation of liver, this chronic inflammation will lead to endothelial cell damage and dysfunction. Oxidative stress is caused by excessive production of ROS or insufficient antioxidant capacity, and this oxidative stress environment further damages endothelial cells (19).

In cirrhotic portal hypertension, there is a reduction in NO synthesis and release. Liver endothelial cells synthesize NO via endothelial nitric oxide synthase (eNOS) in healthy liver, a potent vasodilator that maintains vascular tone and normal blood flow (20). However, eNOS expression and activity are significantly reduced in cirrhosis, leading to reduced synthesis and release of NO (20, 21). The reduced NO production and decreased NO bioavailability directly leads to a decrease in the diastolic ability of intrahepatic blood vessels (21, 22). The blood vessels show continuous contraction, increasing portal system resistance. NO is not only a vasodilator, but also has the effect of inhibiting platelet adhesion and aggregation (23). The reduction of NO causes platelets to adhere to and aggregate on the damaged endothelial surface, forming tiny thrombi, further blocking blood vessels and aggravating microcirculatory disorders (6). The microthrombi formation not only increases the intrahepatic blood flow resistance, but may also lead to local ischemia and further tissue damage (24).

In contrast to the decreased NO, vasoconstrictors are significantly increased in liver cirrhosis. Endothelial dysfunction is also manifested by excessive release of vasoconstrictive factors such as endothelin-1 (ET-1). ET-1 is a potent vasoconstriction factor secreted by endothelial cells (13), inducing contraction of vascular smooth muscle cells (VSMCs) and increasing vascular resistance by binding to its receptor (13). In liver cirrhosis, due to endothelial cell damage and stimulation of inflammatory mediators, the expression and release of ET-1 are significantly increased (25), resulting in continuous vasoconstriction and further aggravating of portal hypertension (26).

#### 2.2.2 Contraction of HSCs

Activated HSCs have contractile function similar to smooth muscle cells, which is mainly achieved through cytoskeletal remodeling and actin (*α*-SMA) expression (27). Contractile HSCs can directly increase hepatic sinusoidal resistance and impede blood flow in the portal system (28). α-SMA is a typical myofibroblast marker, and its expression level is closely related to the contractility of HSCs. Through the regulation of intracellular calcium concentration and the interaction of actin-myosin system (29, 30), contractile HSCs can significantly increase the intrahepatic vascular resistance and further lead to portal hypertension (31). Activated HSCs not only have contractile function, but also secrete a variety of vasoconstrictor factors, such as ET-1 and angiotensin II (AngII) (13). These factors act on intrahepatic blood vessels through autocrine and paracrine pathways, further promoting vasoconstriction and fibrosis (32, 33).

#### 2.2.3 Vasoactive substance imbalance

There is an imbalance between NO and ET-1 in cirrhotic portal hypertension. The reduction of NO and the increase of ET-1 in patients with cirrhosis are typical manifestations of vasoactive substance imbalance (34). The decrease of NO leads to weakened vasodilation, while the increase of ET-1 leads to enhanced vasoconstriction (32). This imbalance between vasodilation and contraction directly leads to increased intrahepatic vascular resistance and increased portal pressure (35).

The expression of prostacyclin (PGI2) and cyclooxygenase-2 (COX-2) is also decreased in liver cirrhosis. PGI2 is a potent vasodilator that inhibits the VSMCs contraction by activating adenylate cyclase (AC) to produce cyclic adenosine phosphate (cAMP) (36). In cirrhosis, the synthesis and release of PGI2 are reduced, resulting in reduced vasodilation (37, 38). COX-2 is a key enzyme in the PGI2 synthesis, and its reduced expression directly affects PGI2 production, further weakening the vasodilation ability (39).

The role of AngII and Angiotensin-converting enzyme (ACE) in the pathophysiology of cirrhotic portal hypertension should not be overlooked. ACE is a key enzyme for AngII production, and its increased activity leads to increased AngII levels (40). AngII is a potent vasoconstriction that causes VSMCs to contract and increase vascular resistance by binding to AngII receptor (41). In addition, AngII also has a pro-fibrotic effect, further aggravating liver fibrosis and portal hypertension by stimulating the activation of HSCs and the production of ECM (42). Inhibiting AngII expression can decrease collagen synthesis (42, 43).

The compensation of vasodilators is obviously insufficient. In cirrhosis, although the levels of certain vasodilator factors such as adrenomedullin (AM) and brain natriuretic peptide (BNP) are elevated to certain extent in an attempt to counteract the overexpression of vasoconstrictors (44, 45), their compensatory effects are often insufficient to maintain normal vascular tone and balance. AM has a strong vasodilatory effect by increased cAMP generation (46). BNP inhibits VSMCs contraction by increasing the production of cyclic guanosine phosphate (cGMP) (47). However, in cirrhosis, the compensatory mechanisms of these vasodilator factors are unable to fully offset the overexpression of vasoconstrictor, resulting in vascular tone imbalance and increased portal pressure.

## 3 Extrahepatic vascular changes

Portal hypertension in cirrhosis leads to major changes in the extrahepatic vascular and systemic circulation. These changes include increased portal blood flow, splanchnic vasodilation, portal-systemic collateral formation, hyperdynamic circulation, and abnormalities in the intestinal and pulmonary microcirculation. Complex molecular mechanisms involve angiogenesis, vasodilation, and oxidative stress (Figure 1).

### 3.1 Extrahepatic portal vascular changes

Portal hypertension is one of the core pathological changes in liver cirrhosis. It is caused by many factors, among which changes of extrahepatic portal vein are particularly critical. These changes mainly include a significant increase in portal blood flow and the formation of portosystemic collateral circulation.

Extrahepatic vascular changes in liver cirrhosis include portal blood flow and systemic hemodynamics. Due to the dilation of visceral blood vessels, especially in the gastrointestinal and splenic region, splanchnic blood flow increases, eventually leading to increased blood flow to portal vein (48–50). In addition, changes in systemic hemodynamics are particularly critical. Patients with liver cirrhosis are usually accompanied by a hypovolemic state and reduced effective blood volume in the systemic circulation (51). This state activates the adrenal glands and the sympathetic nervous system, causing systemic vasoconstriction, especially in the renal and splenic vessels, thereby reducing blood flow elsewhere and increasing portal blood flow (52). At the same time, activation of the renin-angiotensin system by the kidney increases the release of vasoconstrictor factors, which further promotes an increase in portal blood flow. Alterations in splanchnic vasculature and systemic hemodynamics in cirrhosis interact to result in a significant increase in portal blood flow (49).

As portal vein pressure continues to rise, portosystemic collaterals are formed to relieve the pressure (53). Although this compensatory mechanism helps to reduce portal vein pressure in the short term, its long-term consequences can lead to a series of complications, including esophageal and gastric varices, hypersplenism, etc. (54). The vascular regulation mechanisms in this process involves are complex. Firstly, one of the core mechanisms of portosystemic collateral circulation is angiogenesis. In cirrhotic portal hypertension, multiple factors induce overexpression of angiogenic factors such as vascular endothelial growth factor (VEGF) and PDGF (55–57). These factors initiate the formation of new collateral circulation by promoting the proliferation and migration of vascular endothelial cells (56). Hypoxia-inducible factor (HIF-1α) is another key regulator. HIF-1α, stimulated by hypovolemia, not only induces VEGF expression, but also promotes other related angiogenic factors production, further accelerating the generation of collateral circulation (58, 59). Secondly, remodeling of existing blood vessels is also a mechanism that cannot be ignored during the formation of portosystemic collateral circulation. Especially in the esophagus and fundus of the stomach, the original microvascular network expands and remodels driven by portal pressure, forming functional varicose veins (60). Smooth muscle cells and collagen deposition in the blood vessel wall increase, which enhances the capacity of the blood vessel and allows greater blood flow to pass through (61). At the same time, this vascular remodeling process is accompanied by thinning of the vessel wall, increasing the risk of varicose vein rupture bleeding (57, 61). In addition, inflammatory factors not only promote angiogenesis, but also accelerate local blood vascular remodeling (57). At the same time, leukocyte infiltration will also accelerate blood vessels dilation and the formation of collateral circulation.

### 3.2 Changes in systemic circulation

Splanchnic vasodilation is one of the core features of systemic circulatory changes in patients with cirrhotic portal hypertension (62). Splanchnic vasodilation causes a series of adverse consequences, including hypovolemia, ascites and so on (7). In cirrhosis, visceral vascular endothelial cells are dysfunctional, and the levels of vasodilators such as NO, PGI2, and carbon monoxide (CO) are significantly increased (63–65). These factors are mainly produced by endothelial cells. Increased production of vasodilator synthase promotes vascular smooth muscle relaxation and leads to vasodilation (63, 66). In addition, vasoconstrictor factors are relatively reduced. Although vasoconstrictor factors such as ET-1 also increase, their effects are offset by a large number of vasodilator factors. Moreover, the responsiveness of vascular smooth muscle to contractile stimuli decreases (67). Long-term exposure to high concentrations of vasodilator factors reduces the sensitivity of VSMCs to normal contractile stimulation (68). Inflammatory responses also participate in visceral vasodilation and increase vascular permeability, leading to ascites and tissue edema.

Hyperdynamic circulation is another important feature of cirrhotic patients, which is closely related to splanchnic vasodilation and the formation of portosystemic collateral circulation (49). The main manifestations of hyperdynamic circulation include increased cardiac output, decreased peripheral vascular resistance and decreased renal blood flow (69, 70). The occurrence of hyperdynamic circulation involves multiple mechanisms. The first is increased systemic NO production. NO not only causes splanchnic vasodilation, but also reduces arteriolar and capillary resistance through systemic vasodilation, thereby increasing cardiac output (71). This compensatory mechanism is to maintain the oxygen supply requirements of peripheral tissues. Secondly, the sympathetic nervous system and renin-angiotensin system activation are also involved in this process (72). In response to the decrease in systemic vascular resistance, the sympathetic nervous system and renin-angiotensin system are activated to increase blood volume by constricting blood vessels and retaining sodium and water (73). However, this compensatory mechanism has limited effect under the action of NO and other vasodilator factors, and instead aggravates vasodilation and reduce renal blood flow (74). In addition, the role of systemic inflammation in this process cannot be ignored (70). Inflammatory factors promote fluid exudation by increasing vascular permeability, leading to aggravation of ascites. Finally, the compensatory response of the heart will cause some adverse consequences. Long-term compensatory load increase may lead to cirrhotic cardiomyopathy, in which the heart is unable to maintain normal function under increased load (75, 76).

### 3.3 Microcirculation changes

The microcirculation, as the most subtle component of the vascular system, includes small arteries, capillaries and small veins, responsible for the transport of oxygen and nutrients and the discharge of waste. Cirrhotic portal hypertension has a profound impact on the extrahepatic microcirculation such as intestinal tract and lung through complex mechanisms.

The abnormalities of intestinal microcirculation in patients with cirrhosis are mainly manifested in intestinal ischemia, obvious vascular congestion in the intestinal wall, increased vascular permeability, and resulting in intestinal wall edema (77–79). It affects the absorption of nutrients and aggravates the malnutrition of patients (78). In addition, portal hypertension aggravates local intestinal inflammatory response, destroy intestinal barrier function, increase the risk of bacterial translocation, and thus induce systemic inflammatory response syndrome (SIRS) (80). It also promotes the entry of intestinal endotoxins into the portal vein system, aggravating liver inflammation and fibrosis (81). The mechanism of abnormal intestinal microcirculation involves multiple aspects. Firstly, portal hypertension causes intestinal venous congestion, vasodilation, and slow blood flow, leading to insufficient microcirculatory perfusion (79). Secondly, there is an imbalance of vasoactive substances. Increased vasoconstrictor factors, such as ET-1, lead to intestinal microvascular spasm and further aggravate ischemia (82). The decreased bioavailability of NO leads to vascular endothelial dysfunction and further deteriorates intestinal microcirculation (83). In addition, the oxidative stress response is enhanced in cirrhosis, leading to endothelial damage, weakening the dilation ability of blood vessels, and ultimately affecting microcirculatory function (84).

Changes in pulmonary microcirculation are a common but easily overlooked complication in patients with cirrhotic portal hypertension. In terms of pulmonary microcirculation, the main manifestations are hepatopulmonary syndrome and hepatogenic pleural effusion. Hepatopulmonary syndrome is characterized by abnormal dilation of pulmonary blood vessels and redistribution of intrapulmonary blood flow (85), leading to oxygenation dysfunction (86); while hepatogenic pleural effusion is caused by obstruction of lymphatic drainage. In contrast to hepatogenic pleural effusion, hepatic hydrothorax develops when ascitic fluid moves from the peritoneal cavity into the pleural space through diaphragmatic defects (87), which is unrelated to pulmonary microcirculatory disorders. The mechanism of pulmonary microcirculation changes involves multiple aspects. The first is that pulmonary vasodilation occurs primarily at the level of alveolar capillaries. In liver cirrhosis, excessive synthesis of NO will cause abnormal expansion of pulmonary capillaries, increasing pulmonary blood flow, but decreasing oxygen diffusion efficiency, resulting in hypoxemia (88). In addition, the exchange time of oxygen between the alveoli and blood is insufficient, resulting in gas exchange disorder (89, 90). Increased arteriovenous shunting also leads to alveolar ventilation-blood flow imbalance (91). The chronic inflammatory response and oxidative stress will increase the permeability of pulmonary capillaries, leading to pulmonary edema and further aggravate the damage of lung function (90, 92, 93). Recent studies have revealed that increased pulmonary expression of placental growth factor (PlGF) and VEGF-A plays a central role in pathological angiogenesis (94). The von Willebrand factor-angiopoietin axis activation and altered circadian rhythm proteins, particularly BMAL1, significantly affect hypoxic responses and vascular remodeling (88, 95). Additionally, bacterial translocation and endotoxemia contribute to pulmonary inflammation through recruitment of intravascular monocytes that produce proangiogenic factors (96). These molecular mechanisms create extensive pulmonary microvascular alterations, including capillary dilatation, arteriovenous malformations, and altered vascular reactivity. In contrast, portopulmonary hypertension (POPH) represents a distinct entity characterized by pulmonary arterial hypertension in the setting of portal hypertension (97). The pathophysiology involves pulmonary vasoconstriction, vascular remodeling, and *in situ* thrombosis. Key molecular pathways include endothelial dysfunction with decreased NO and prostacyclin production, upregulation of ET-1 and serotonin pathways, and proliferation of pulmonary arterial smooth muscle cells (98). BMP9 is a sensitive and specific biomarker of POPH, which could predict transplant-free survival and the presence of pulmonary arterial hypertension in liver disease (99). The mechanical stress from increased pulmonary blood flow in the hyperdynamic circulatory state may trigger endothelial injury, initiating these pathological cascades.

## 4 Pharmacological interventions based on intrahepatic vascular changes

Intrahepatic vascular changes play a role in liver cirrhosis. And therapeutic strategies targeting intrahepatic vessels mainly focus on anti-fibrotic treatment, improvement of hepatic sinusoidal microcirculation, and treatment targeting HSCs (Table 1).

**Table 1:** Evidence for pharmacological therapy targeting intrahepatic and extrahepatic vascular dysregulations in portal hypertension.

	Categories	Agent	Model	Research subjects	Direct target	Mechanism
Pun et al. (143)	Clinical drugs	Fructooligos-accharides	BDL rats	Intrahepatic vessels	ROS/eNOS	Ameliorate of dysbiosis and oxidative stress
Asada et al. (144)		Tofogliflozin	CCl_4_ rats	LSECs, HSCs	SGLT 2 inhibitors	Improving endothelial dysfunction
Fan et al. (57)		Cediranib	BDL rats	Intrahepatic and extrahepatic vessels	VEGFR-2	Improving vascular remodeling and contractility
Vairappan et al. (145)		Candesartan cilexetil	CCl_4_ mice	HUVECs	Nostrin-eNOS-NO	Improving endothelial dysfunction
Tai et al. (146)		Celecoxib	TAA rats	LSECs	eNOS	NO homeostasis
Noah et al. (147)		Empagliflozin	CCl4 rats	LSECs, HSCs	Gal-1/NRP-1	Suppression of angiogenesis
Zheng et al. (148)		Telmisartan	CCl4, BDL rats	Liver and mesenteric tissue	KLF-4, eNOS	Reducing angiogenesis and vascular remodeling
Zhu et al. (67)	Small molecular agents	8-OH-DPAT	TAA, BDL, PPVL rats	VSMCs	5-HT receptor 1A	Inducing the contraction of portal vein
Zhao et al. (61)		Imperatorin	CCl_4_ rats	HSCs	TGF-β	Reducing hepatic fibrosis and vascular remodeling
Li et al. (149)		Urolithin A	CCl_4,_ BDL mice	HSCs	Glutaminas-e1	Inhibiting fibrogenesis and HSCs contraction
Gunarathne et al. (150)		MrgD	BDL, PPVL, CCl_4_ rats	Splanchnic vessels	Mas receptor	Mesenteric Vasodilation
Wang et al. (151)		DPP4i	CCl_4_ rats	Mesenteric arterioles	Nox4	Normalizing arterial hypocontractility
Pun et al. (152)		Glycyrrhizin	BDL rats	Mesenteric vessels	VEGF	Attenuating portosystemic collateral shunting
Boyer-Diaz et al. (153)		Lanifibranor	TAA, BDL rats	HSCs, LSECs	Pan-PPAR agonist	Ameliorating hepatic microvascular function
Brusilovskaya et al. (154)		TADA	BDL rats	Intrahepatic vessels	PDE-5 inhibitor	Reducing sinusoidal vascular resistance
Tsai et al. (155)		Obeticholic acid	BDL rats	Intrahepatic vessels	Farnesoid X receptor agonist	Inhibiting vasoconstriction
Hu et al. (156)		AICAR	BDL, PPVL, CCl_4_ rats	LSECs	AMPK/NO	Improving NO bioavailability
Castillo (157)		PHIN-156	BDL rats	Vasopressin receptor	V1a partial agonists	Reducing portal blood flow
Jones (158)		BI 685509	TAA rats	Portosystemic shunting	sGC, cGMP	NO-independent sGC activator
Zhao et al. (39)		PTUPB	CCl4 rats	Intrahepatic and extrahepatic vessels	sEH/COX-2/TGF-β	Inhibiting intra-or extrahepatic angiogenesis and vascular remodeling

BDL, bile duct ligation; eNOS, endothelial nitric oxide synthase; ROS, reactive oxygen species; CCl_4,_ Carbon tetrachloride; LSECs, liver sinus endothelial cells; HSCs, hepatic stellate cells; SGLT2, Sodium glucose transferase 2; VEGFR-2, vascular endothelial growth factor receptor 2; HUVECs, human umbilical vein endothelial cells; NO, Nitric oxide; TAA, thioacetamide; Gal-1, galactin-1; NRP-1, Neuropilin-1; KLF-4, Krüppel-like factor-4; PPVL, partial portal vein ligation; VSMCs, vascular smooth muscle cells; 5-HT, 5-hydroxytryptamine; TGF-β, Transforming growth factor-β; MrgD, Mas-related G protein-coupled receptor type D; DPP4i, Dipeptidyl peptidase-4 inhibitor; Nox4, NADPH oxidase 4; VEGF, vascular endothelial growth factor; Pan-PPAR, pan-peroxisome proliferator-activated receptor; TADA, Soluble guanylyl cyclase stimulation and phosphodiesterase-5; PDE-5, phosphodiesterase-5; AICAR, 5-aminoimidazole-4-carboxyamide ribonucleoside; AMPK, Adenosine 5′-monophosphate-activated protein kinase; V1a, vasopressin 1a; sGC, soluble guanylyl cyclase; cGMP, cyclic guanosine monophosphate; PTUPB, 4-(5-phenyl-3-57-pyrazol-1-yl) -benzenesulfonamide; sEH, soluble epoxide hydrolase; COX-2, cyclooxygenase-2.

### 4.1 Antifibrotic therapy

Antifibrotic therapy is one of the cornerstones of cirrhotic portal hypertension treatment. Liver fibrosis leads to structural remodeling and functional abnormalities of intrahepatic blood vessels, thereby causing increased portal pressure. Therefore, inhibiting and reversing the process of liver cirrhosis has become a key strategy to reduce portal pressure.

Firstly, inhibiting excessive deposition of ECM. The main pathological feature of liver cirrhosis is the massive ECM deposition (100). Antifibrotic treatments aim to reduce or reverse the accumulation of ECM (3). Many drugs and molecular targets can intervene in this process, including blocking liver fibrosis formation by inhibiting the TGF-*β* signaling pathway (14). TGF-β is an important profibrotic factor in liver fibrosis, and inhibiting its activity can significantly reduce the degree of fibrosis. Activators of matrix metalloproteinases (MMPs) can promote ECM degradation (101); while inhibitors of tissue inhibitors of metalloproteinases (TIMPs) can reduce ECM deposition (102). Therefore, MMP/TIMP balance is a potential therapeutic target for regulating the extracellular matrix (102, 103). Activation of HSCs can lead to imbalances in MMP2/TIMP2 and MMP9/TIMP1, aggravating fibrosis (104, 105).

Secondly, applying antioxidant and anti-inflammatory treatment. Oxidative stress and inflammatory responses also play a key role in the process of liver cirrhosis. Therefore, antioxidants and anti-inflammatory drugs are used to alleviate oxidative stress damage to liver cells. Common antioxidants include vitamin E, lipoic acid, etc., which improve liver fibrosis by reducing the ROS production (106, 107). Wang Q, et al. found that glycyrrhizic acid inhibited oxidative stress injury through targeting AKR7A2 in HSCs, reduced the activated HSCs proliferation and reversed hepatic fibrosis (108). In addition, anti-inflammatory drugs such as glucocorticoids and certain immunomodulators can reduce the chronic inflammatory response and progression of liver cirrhosis. Qin BF, et al. found that specnuezhenide inhibited inflammatory response via SIRT6-P2X7R/NLRP3 pathway and improve fibrosis (109).

Targeting HSCs activation is important in pharmacological treatments for patients with cirrhotic portal hypertension. Activated HSCs are the main effector cells of liver cirrhosis. By inhibiting the activation of HSCs or promoting their apoptosis, ECM production can be effectively reduced (110). Some drugs, such as retinoic acid receptor gamma agonists (such as retinoic acid) and peroxisome proliferator-activated receptor gamma (PPARγ) agonists, have shown the potential to inhibit HSCs activation (111). Benedicto AM, et al. have shown that interference with mitochondrial function could target HSCs to inhibit fibrosis (110). Tung HC, et al. demonstrated that inhibition of heme-thiolate monooxygenase CYP1B1 could decrease HSCs activation and fibrosis (12).

Promoting hepatocyte regeneration can improve liver function and indirectly reduce fibrosis. Growth factors such as hepatocyte growth factor (HGF) and epidermal growth factor (EGF) have been shown to promote liver regeneration and reduce fibrosis. Wang P, et al. have identified hepatic Snai1 and Snai2 as key transcriptional regulators of liver regeneration and fibrosis (112). Novel stem cell therapies, such as mesenchymal stem cells (MSCs), possess immunomodulatory and anti-inflammatory capabilities that make them an attractive approach for promoting liver regeneration (113). The primary mechanism involves promoting apoptosis of HSCs and subsequently stimulating hepatocyte proliferation, thereby replacing damaged hepatocytes and reducing liver fibrosis (114).

### 4.2 Improvement of hepatic sinusoidal microcirculation

Hepatic sinusoidal microcirculation disorder is one of the important mechanisms for portal hypertension. It can directly reduce intrahepatic vascular resistance, thereby reducing portal pressure.

Statins, the most widely used lipid-lowering drugs, have been found in recent years to have the potential to improve liver cirrhosis and hepatic sinusoidal microcirculation (115, 116). Statins increase the expression and activity of eNOS, promoting the production of NO, and reducing hepatic sinusoidal resistance (116). In addition, statins can inhibit the production of inflammatory factors, such as TNF-*α* and IL-6, thereby reducing microcirculation disorders caused by inflammation (117). Statins can also protect LSECs and maintain their normal function by reducing the ROS production (117, 118). Statins can also reduce hepatic sinusoidal contraction and improve microcirculation by inhibiting Rho kinase activity (119). Some studies have shown that simvastatin and atorvastatin can significantly reduce portal pressure and improve the prognosis of patients with cirrhosis (120, 121). However, it should be noted that statins should be used with caution in patients with advanced cirrhosis to avoid potential hepatotoxicity (122). Statins represent the most clinically advanced antifibrotic therapy, with multiple Phase III trials demonstrating their potential in portal hypertension. Simvastatin and atorvastatin have shown particular promise, with data supporting their safety in compensated cirrhosis. However, their use in advanced cirrhosis requires careful monitoring.

In liver cirrhosis, NO production is reduced due to endothelial cell dysfunction, leading to increased vasoconstriction. Therefore, exogenous NO donors, such as nitrates, can improve the expansion ability of liver sinuses and reduce intrahepatic vascular resistance (32). In addition, NO donors can inhibit the contraction of HSCs and reduce their compression on hepatic sinusoids, thereby improving microcirculation (20). Villanueva C, et al. have found that isosorbide mononitrate (ISMN) can significantly reduce portal pressure and prevent variceal rebleeding, especially during acute application (123). However, long-term use may lead to the reduced tolerance and effectiveness of treatment. To overcome tolerance issues, researchers are exploring intermittent dosing regimens and novel NO donors. For example, NCX-1000 is a liver-targeted NO donor that can specifically release NO, promising to improve therapeutic efficacy and reduce systemic side effects (124). In addition, combination treatment strategies combining statins and nitrates also show good promise. This combination can work synergistically through different mechanisms to improve hepatic sinusoidal microcirculation more effectively. Nicorandil and atorvastatin may alleviate hepatic sinusoidal microcirculatory disorders by improving liver function, anti-inflammation and anti-oxidation (125).

Endothelin (ET) receptor antagonist is another choice of hepatic sinusoidal microcirculation improvement. There is an observed up-regulation of the ET-1 gene accompanied by a compensatory down-regulation of the ET A receptor (ETAR) gene in the human portal vein (25). Blocking the ET-1/ETAR pathway using selective ETAR antagonists (ERAs) represents a promising therapeutic strategy for liver cirrhosis treatment (26). Ten Hove M, et al. have demonstrated that engineered SPIONs functionalized with ETAR antagonist had improved liver fibrosis through the inhibition of HSCs activation (13). A selective ET-A antagonists, such as BQ 123 and Ambrisentan, decrease the portal pressure in cirrhotic patients (126).

### 4.3 Treatment targeting the contractile function of HSCs

HSCs play a central role in the development of portal hypertension. Activated HSCs are not only the main ECM producers that lead to liver fibrosis, but also have contractile properties and are directly involved in the regulation of hepatic sinusoidal resistance. Therefore, therapeutic strategies targeting HSCs have become a hot topic in recent years. HSCs have contractile properties and are directly involved in the regulation of liver sinusoidal resistance (127). ET-1 is a potent vasoconstrictor that can cause HSCs to contract. The use of ET-1 receptor antagonists can reduce HSCs contraction and reduce liver sinusoidal resistance (25). In addition, AngII can promote HSCs contraction and proliferation (128). AngII receptor antagonists, such as losartan, can reduce HSCs contraction and improve liver sinusoidal microcirculation (42). Moreover, Nanotechnology can be used to achieve targeted drug delivery, improve therapeutic effects and reduce side effects. Vitamin A-modified liposomes can specifically deliver drugs to HSCs because HSCs are the main vitamin A storage cells in liver. This strategy can be used to deliver anti-fibrotic drugs, siRNA or gene therapy vectors. Kaili Wang et al. constructed hyaluronic acid (HA) modified liposomes co-delivering all-trans retinoic acid (RA) and L-arginine (L-arg) to reverse hepatic fibrosis (129). Lingfeng Zhang et al. designed chondroitin sulfate-modified and vismodegib-loaded nanoparticles (CS-NPs/VDG) to efficiently normalize the fenestrae phenotype of LSECs and restore HSCs to quiescent state by inhibiting Hedgehog signaling pathway (130). Additionally, stem cell therapy is a treatment method targeting HSCs that has attracted much attention in recent years. MSCs have multidirectional differentiation potential and immunomodulatory functions, and can inhibit HSCs activation by secreting various anti-inflammatory and anti-fibrotic factors (131, 132). Preliminary clinical studies have shown that stem cell therapy is effective in reducing liver fibrosis and improving liver function (131). These have inspired new ways of thinking about treating liver fibrosis.

## 5 Pharmacological interventions based on changes in extrahepatic vessels

Extrahepatic vascular dilation, increased blood flow, and changes in peripheral vascular resistance exacerbate the portal pressure. Therefore, treatment strategies based on changes in extrahepatic blood vessels aim to reduce portal blood flow, regulate vascular tension, and improve systemic hemodynamic balance (Table 1). The clinical development of therapies targeting extrahepatic vascular changes shows a clear stratification in terms of evidence and approval status. Non-selective beta blockers (NSBBs) and vasoconstrictors represent the current standard of care, supported by Class I, Level A evidence. Propranolol, carvedilol, and nadolol are FDA-approved and widely used in clinical practice. For acute complications, terlipressin and octreotide have established roles in management protocols. Beyond these approved therapies, several novel approaches are in various stages of clinical development. Understanding this therapeutic hierarchy is essential for optimal clinical decision-making and future research directions.

### 5.1 NSBBs

NSBBs are classic drugs for cirrhotic portal hypertension (133). The main mechanisms include reducing cardiac output by blocking β1 receptors, and causing splanchnic vasoconstriction and reducing portal blood flow by blocking β2 receptors. NSBBs can effectively reduce the burden on portal system, and are especially suitable for preventing esophageal and gastric variceal bleeding (134). Propranolol and Carvedilol are commonly used NSBBs. These drugs are widely used for the primary prevention of portal hypertension, which is to prevent bleeding from varicose veins that are not yet bleeding (135). For patients who have already suffered bleeding, NSBBs are also used for secondary prevention to reduce the risk of rebleeding (136). Although NSBBs are effective in reducing portal pressure and preventing variceal rupture, not all patients can tolerate these drugs, especially those with hypotension or severe cardiac dysfunction (137). In addition, NSBBs may interact with other medications, so they should be used with caution.

### 5.2 Vasoconstrictors

Vasoconstrictors reduce portal pressure by constricting the visceral arterial system and reducing blood flow to the portal vein. Their main target is the visceral vascular smooth muscle, directly or indirectly regulating its contractile function. They are usually used to treat acute complications of portal hypertension, such as gastric variceal. Terlipressin, one of the most commonly used vasoconstrictors clinically, reduces portal vein blood flow by selectively acting on V1 receptors in visceral blood vessels (138). Long-term continuous infusion of terlipressin can significantly increase cardiac reserve and attenuate a hyperdynamic state (139). Octreotide have similar effect for the management of variceal bleeding (140). The main side effects of vasoconstrictors include increased blood pressure, myocardial ischemia, and impaired renal function. Therefore, patients with cardiovascular disease or renal insufficiency should be used with extreme caution and closely monitored. In addition, long-term use of these drugs may lead to decreased renal perfusion and increase the risk of AKI (141).

### 5.3 Angiogenesis inhibition therapy

An important feature of cirrhotic portal hypertension is abnormal angiogenesis in the visceral vascular system, especially in the spleen and intestinal areas. These abnormal neovascularization structures are unstable and permeable, leading to increased portal vein pressure. Therefore, targeted treatment strategies to inhibit abnormal angiogenesis have gradually become the focus of research. Cediranib may ameliorate extrahepatic hyperdynamic circulation by targeting angiogenesis. This is achieved through the inhibition of vascular endothelial growth factor receptor 2 (VEGFR-2) signaling, thereby reducing both portal collateral vessel formation and eNOS-mediated vasodilation and vascular remodeling (57). Hydroxysafflor yellow A is a multi-target tyrosine kinase inhibitor that inhibits the VEGF and PDGF signaling pathways, thereby inhibiting abnormal angiogenesis (142). Although anti-angiogenic therapy has great potential in theory, its clinical application is still being explored. These drugs may cause systemic side effects such as hypertension, bleeding, and delayed wound healing, so they should still be used with caution.

## 6 Future prospective

Future research will focus on further elucidating the intricate intrahepatic and extrahepatic vascular regulatory mechanisms underlying portal hypertension in cirrhosis.

- Nanotechnology for Targeted Therapies

Emerging nanotechnology offers new possibilities for the treatment of portal hypertension. Utilizing nanocarriers enables precise drug delivery to specific cells or tissues, such as HSCs or LSECs. This not only enhances drug concentration at the site of action but also minimizes off-target effects on healthy tissues. This strategy holds significant promise for improving both the efficacy and safety of therapeutic interventions. However, nanotechnology approaches are currently in preclinical development and require additional safety data.

- The Promise of Stem Cell Therapy

Stem cell therapy, a burgeoning therapeutic modality, has demonstrated potential in early clinical trials for reducing liver fibrosis. MSCs, through their immunomodulatory and anti-inflammatory properties, can suppress HSC activation and reduce ECM production, thereby slowing the progression of fibrosis. Future research will further investigate the long-term efficacy of stem cell therapy in individuals with cirrhosis and explore strategies to enhance stem cell functionality through gene editing techniques. Stem cell therapies, while showing promise in animal studies, are still in early development phases. MSCs has progressed to Phase I trials, focusing primarily on safety assessments in cirrhotic patients.

- Optimization of Existing Drugs and Development of Novel Agents

Future research will focus on optimizing the efficacy and safety of existing drugs. For example, while statins have shown promise in improving sinusoidal microcirculation and reducing portal pressure, their long-term safety requires further validation. The development of novel agents targeting pathological mechanisms like angiogenesis and vasodilation will also be a priority. Although anti-angiogenic drugs, such as VEGF inhibitors, are effective, they can cause systemic side effects like hypertension. Therefore, research will focus on improving drug targeting and minimizing adverse reactions. The safety and preliminary efficacy of novel NO donors are in the early clinical development stage. New small molecules targeting specific pathways require toxicology studies. VEGF inhibitors, obeticholic acid, and rifaximin combined with statins have entered the clinical research stage.

## 7 Conclusion

Cirrhotic portal hypertension involves complex intrahepatic and extrahepatic vascular mechanisms. Comprehensive treatments such as improving microcirculation and regulating vascular tension can effectively reduce portal pressure, alleviate complications, and improve patient prognosis. More research is needed in the future to validate drugs targeting intrahepatic and extrahepatic vascular disorders in order to improve treatment of portal hypertension.
